# Associations between Specific Redox Biomarkers and Age in a Large European Cohort: The MARK-AGE Project

**DOI:** 10.1155/2017/1401452

**Published:** 2017-07-19

**Authors:** Daniela Weber, Wolfgang Stuetz, Olivier Toussaint, Florence Debacq-Chainiaux, Martijn E. T. Dollé, Eugène Jansen, Efstathios S. Gonos, Claudio Franceschi, Ewa Sikora, Antti Hervonen, Nicolle Breusing, Thilo Sindlinger, María Moreno-Villanueva, Alexander Bürkle, Tilman Grune

**Affiliations:** ^1^Department of Molecular Toxicology, German Institute of Human Nutrition Potsdam-Rehbruecke (DIfE), 14558 Nuthetal, Germany; ^2^NutriAct-Competence Cluster Nutrition Research Berlin-Potsdam, 14458 Nuthetal, Germany; ^3^Institute of Biological Chemistry and Nutrition, University of Hohenheim, 70599 Stuttgart, Germany; ^4^URBC-NARILIS, University of Namur, 5000 Namur, Belgium; ^5^National Institute of Public Health and the Environment (RIVM), 3720 BA Bilthoven, Netherlands; ^6^Institute of Biological Research and Biotechnology, National Hellenic Research Foundation (NHRF), 11635 Athens, Greece; ^7^Department of Experimental Pathology, University of Bologna, 40126 Bologna, Italy; ^8^Nencki Institute of Experimental Biology, Polish Academy of Sciences, 02-093 Warsaw, Poland; ^9^School of Medicine, University of Tampere, 33014 Tampere, Finland; ^10^Department of Applied Nutritional Science/Dietetics, Institute of Nutritional Medicine, University of Hohenheim, 70599 Stuttgart, Germany; ^11^Molecular Toxicology, Department of Biology, University of Konstanz, 78457 Konstanz, Germany; ^12^German Center for Diabetes Research (DZD), 85764 Munich-Neuherberg, Germany; ^13^German Center for Cardiovascular Research (DZHK), 13357 Berlin, Germany

## Abstract

Oxidative stress and antioxidants play a role in age-related diseases and in the aging process. We here present data on protein carbonyls, 3-nitrotyrosine, malondialdehyde, and cellular and plasma antioxidants (glutathione, cysteine, ascorbic acid, uric acid, *α*-tocopherol, and lycopene) and their relation with age in the European multicenter study MARK-AGE. To avoid confounding, only data from countries which recruited subjects from all three study groups (five of eight centers) and only participants aged ≥55 years were selected resulting in data from 1559 participants. These included subjects from (1) the general population, (2) members from long-living families, and (3) their spouses. In addition, 683 middle-aged reference participants (35–54 years) served as a control. After adjustment for age, BMI, smoking status, gender, and country, there were differences in protein carbonyls, malondialdehyde, 3-nitrotyrosine, *α*-tocopherol, cysteine, and glutathione between the 3 study groups. Protein carbonyls and 3-nitrotyrosine as well as cysteine, uric acid, and lycopene were identified as independent biomarkers with the highest correlation with age. Interestingly, from all antioxidants measured, only lycopene was lower in all aged groups and from the oxidative stress biomarkers, only 3-nitrotyrosine was increased in the descendants from long-living families compared to the middle-aged control group. We conclude that both lifestyle and genetics may be important contributors to redox biomarkers in an aging population.

## 1. Introduction

Oxidative stress and antioxidants have been in the focus of research for decades due to their association with numerous age-related diseases [1–4]. In humans, elevated levels of oxidative stress have been reported in several medical conditions, including neurodegenerative diseases [2], obesity, diabetes mellitus, and the aging process itself [3–5]. There is some epidemiological evidence on the role of oxidative stress in aging, age-related diseases, and mortality [6–10]. However, these studies focused mostly on single biomarkers or on biomarkers which are not widely used, thus making it difficult to compare result.

When attempting to assess redox biomarkers, it is important to note that there is not one single biomarker which is considered a “gold standard.” In fact, it is recommended to measure a set of different biomarkers [11]. Thus, our attempt was to analyze markers of protein oxidation, nitration, lipid peroxidation, and cellular and plasma antioxidants and study their relation with age in participants of the MARK-AGE project. The MARK-AGE project was a European multicenter study, supported by the European Commission, aiming to identify biomarkers of human aging and to model a robust set of markers of biological age and healthy aging [12].

For this purpose, women and men were recruited from the general population from eight European countries as age-stratified subgroups, as well as subjects belonging to a family with long-living members together with their spouses. In different biological matrices (whole blood, serum, plasma, urine, buccal mucosa cells, and peripheral blood mononuclear cells), a large number of candidate biomarkers of aging, including DNA-based markers, markers based on proteins and their modifications, immunological markers, clinical chemistry, hormones and markers of metabolism, oxidative stress markers, and antioxidant micronutrients were assessed. The project design including details of the study population and standard operating procedures have been published recently [12–15].

The objectives of the present work were to assess and to compare levels of redox biomarkers within the three different study groups of the MARK-AGE project. Our hypotheses were (1) that oxidative stress is elevated in higher age groups as it has been shown in different rather small population-based studies in different biological matrices and (2) that subjects from families with long-living members would be genetically better equipped to handle oxidative stress than the general population. The results of the spouses might show whether (3) a shared lifestyle may be able to influence biomarker concentrations.

## 2. Materials and Methods

This study was conducted in accordance with the Declaration of Helsinki (1964) and with informed written consent of each participant. Ethical clearance had been given by the ethics committee of each of the recruiting centers. This study has been registered retrospectively at the German Clinical Trials Register (DRKS00007713).

### 2.1. Study Population and Sample Collection

For the whole MARK-AGE project, 3158 participants were recruited in 8 recruiting centers and the details for the three study groups will be described briefly below.

The first study group was recruited through various public platforms such as radio and newspaper advertising. These participants were included in the RASIG group (recruited from the age-stratified general population). For this group, the main inclusion criteria were the ability to give informed consent and being in the age-range from 35 to 75 years (both genders).

The second study group consisted of 537 descendants from long-living subjects (nonagenarians; persons who reached the age of 90) who had been recruited as a follow-up from the GEHA study (Genetics of Healthy Aging; 2004–2009, for details, see [16]); this group is abbreviated as GO (GEHA offspring). The third study group consisted of the spouses of the GEHA offspring (*n* = 311) and served as a lifestyle control group, the so-called SGO (spouses of GO). The GO and SGO participants were between the age of 55 and 75 years [12] and were recruited in Belgium, Finland, Greece, Italy, The Netherlands, and Poland.

Collection of anthropometric data, questionnaire data, and data on cognitive function was carried out by trained nurses or physicians between November 2008 and June 2012 at the following recruiting centers: Hall in Tirol/Innsbruck (Austria), Namur (Belgium), Esslingen (Germany), Athens, and other nearby regions (Greece), Bologna (Italy), Warsaw (Poland), Tampere (Finland), and Leiden (The Netherlands). Furthermore, participants were asked to complete questionnaires on lifestyle characteristic (nutrition, smoking habits), family history, and living environment.

To avoid possible confounding, we used only data from those countries which recruited subjects from all three study groups (RASIG, GO, and SGO). This was the case in five of the eight centers (Belgium, Greece, Italy, Poland, and Finland). Germany and Austria did not provide GO or SGO data while The Netherlands did not recruit RASIG participants; therefore, data from these three countries were excluded in the present analyses.

Since the age ranges of participants in the GO and SGO groups were different to the RASIG group in general (55–75 versus 35–75 years, resp.), only participants who were ≥55 years of age were selected. This resulted in a total of 1559 participants in all three study groups to be included in the present work. Additionally, to compare these study groups to a younger (middle-aged) reference group, we selected all RASIG participants aged 35–54 (“middle aged”) from Belgium, Greece, Italy, Poland, and Finland, which resulted in a total of 683 reference participants.

### 2.2. Determination of Total Glutathione and Total Free Cysteine in Whole Blood

Total glutathione and total free cysteine in whole blood were measured as previously described by Chen et al. [17] by using Ellman's reagent (5,5′-dithiobis-[2-nitrobenzoic acid]) after the reduction of any disulfides present. The modifications regarding the reduction agent DTT (1,4-dithiothreitol), the adaption for whole blood samples, and HPLC conditions were as follows: Whole blood (100 *μ*L) was vortex-mixed with DTT (12.5 mM, 100 *μ*L) and incubated for 3 min; cold trichloroacetic acid solution (10% *w*/*v*, 200 *μ*L) was added; and samples were thoroughly mixed and centrifuged at 19,500 ×g for 5 min at 4°C. The clear supernatant (200 *μ*L) was then added to Ellman's reagent (30 mM, 50 *μ*L) together with di-potassium hydrogen phosphate buffer (2 M, 100 *μ*L), and vortex-mixed. Twenty *μ*L was analyzed on a Shimadzu Prominence HPLC (LC-20A) equipped with an UV-Vis detector (SPD-20AV set at 326 nm). The separation of cysteine and glutathione was achieved by using a Reprosil-Pur 120 C18 AQ column (5 *μ*m, 250 mm × 4.6 mm; Dr. Maisch, Germany) set to 40°C and a mobile phase consisting of methanol (15% *v*/*v*) and acetate buffer (0.05 M, pH 5) at a flow rate of 1 mL/min. Standards diluted to physiological concentrations (62.5–250 *μ*M for cysteine and 500–2000 *μ*M for glutathione) and treated as a sample were used for quantification.

### 2.3. Determination of Ascorbic Acid and Uric Acid in Plasma

Plasma ascorbic acid and uric acid were analyzed by RP-HPLC and UV detection after reduction with tris-(2-carboxyethyl)-phosphine [18]. Briefly, plasma (100 *μ*L) was mixed with tris-(2-carboxyethyl)-phosphine (20% *w*/*w*, 25 *μ*L) and incubated for 5 min on ice; then, freshly prepared metaphosphoric acid solution (10% *w*/*w*, 75 *μ*L) was added and vortex-mixed; and samples were centrifuged at 19,500 ×g and 4°C for 10 min. Twenty *μ*L of clear supernatant was analyzed on a Shimadzu Prominence HPLC and using a 5 *μ*m analytical column (Reprosil-Pur 120 C18 AQ, 250 mm × 4.6 mm; Dr. Maisch, Germany) set to 40°C, a mobile phase consisting of 0.05 M sodium phosphate buffer (pH 2.5) at a flow rate of 1 mL/min and UV-Vis detector (SPD-20AV) set to 245 nm. Pure standards diluted to physiological concentrations (2.5–20 mg/L for ascorbic acid and 20–100 mg/L for uric acid) and treated as a sample were used for quantification.

### 2.4. Malondialdehyde

Plasma malondialdehyde was determined by RP-HPLC coupled with fluorescence detection after derivatization with thiobarbituric acid as described by Wong et al. [19] with modifications [20].

### 2.5. Analysis of Protein Carbonyls and 3-Nitrotyrosine

The analyses of protein carbonyls [21] and 3-nitrotyrosine in plasma by in-house ELISA have been described elsewhere [20].

### 2.6. Analysis of *α*-Tocopherol and Lycopene

Plasma lycopene and *α*-tocopherol were analyzed by RP-HPLC coupled with UV-vis and fluorescence detection as previously described [22].

### 2.7. Statistical Analysis

Demographic characteristics are described by using means ± standard deviation (SD) for continuous variables (age, weight, and BMI) and frequencies (%) for categorical variables (gender, smoking status, age groups, and country). Differences in characteristics between age groups and study groups were compared by one-way ANOVA (continuous variables) with Tukey's post hoc test and Pearson's chi-squared test (prevalence; for categorical variables). Data of plasma biomarkers were transformed appropriately to achieve normal distribution using square root (SR) or logarithmic (LN) transformation and are described by geometric means with 95% confidence intervals (CI). Correlations among biomarkers and between biomarkers and age are shown as Pearson product-moment correlation for transformed data. Mean values of plasma biomarkers between study groups were compared using one-way ANOVA and general linear models with Fisher's least significant difference test. The models were adjusted for age, BMI, gender, smoking status (covariates), and country (factor). In addition, a multiple linear regression analysis with all biomarkers in the initial model and a forward stepwise approach was applied to identify independent plasma biomarkers with the highest correlation with age. Differences of concentrations in biomarkers between RASIG, GO, SGO, and age groups (5-year intervals) are presented as box plots. All statistical analyses were carried out using SPSS software (SPSS Inc., Chicago, IL; Version 19); statistical significance for all tests was considered at *P* < 0.05.

## 3. Results

Characteristics of the study groups are shown in Table 1. The mean age of participants was 64.3 ± 5.4 (55–75) years with no significant difference between the matched study groups. Men represented 47.7% of participants. The mean BMI was 27.3 ± 4.5 kg/m^2^ with no statistical significant difference among the study groups. Only 14.8% of participants were current smokers. The prevalence of smoking was different among the three study groups being 9.9% in SGO, 12.6% in GO, and 17.9% in RASIG. The number of participants from each study center was significantly different as shown in Table 1.

A total of 1559 participants were distributed into the groups as follows: RASIG (*n* = 794), GO (*n* = 493), and SGO (*n* = 272).

**Figure 1:**
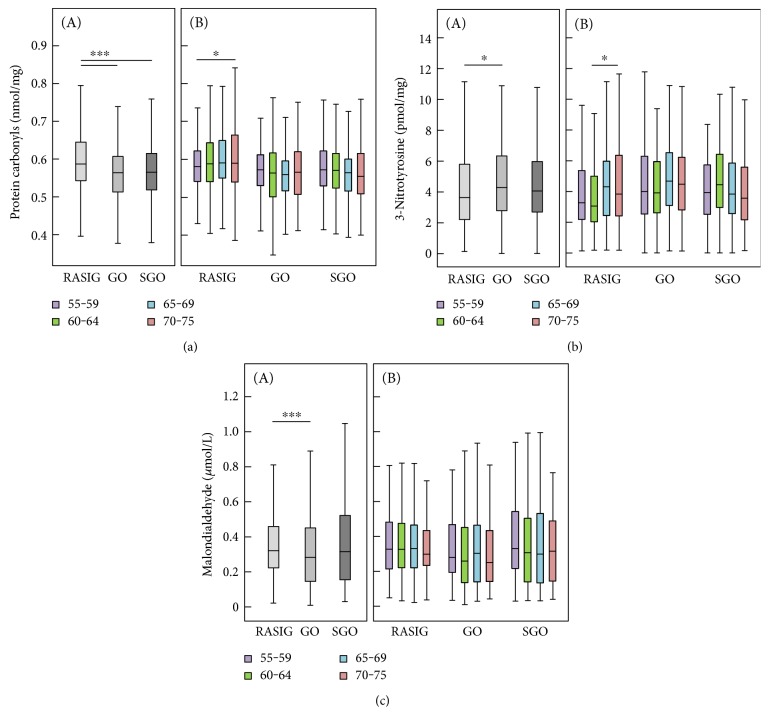
Classical biomarkers of oxidative stress: protein carbonyls (a), 3-nitrotyrosine (b), and malondialdehyde (c). Biomarker concentrations are displayed according to study groups (A) and age groups (B), respectively. RASIG (*n* = 794); GO (*n* = 493); SGO (*n* = 272). Outliers and extreme values are included in the analyses but not shown in the figure. Statistically significant differences are indicated by asterisks: ^∗^*P* < 0.05 and ^∗∗∗^*P* < 0.001.

**Figure 2:**
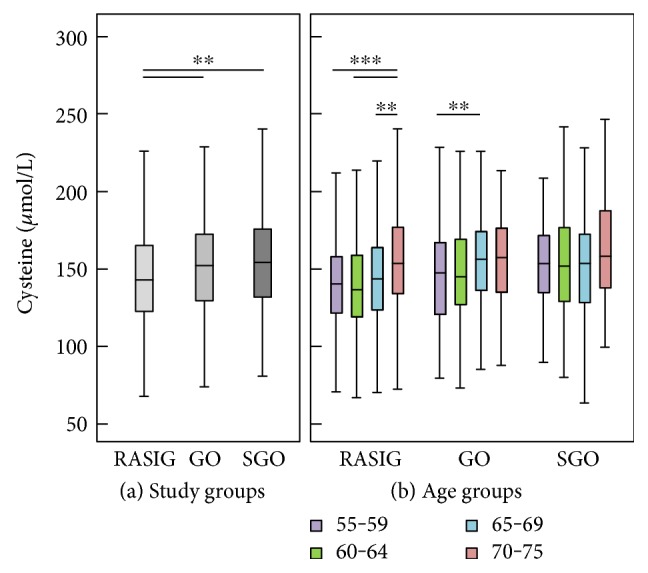
Cysteine concentration by study groups (a) and age groups (b). RASIG (*n* = 794); GO (*n* = 493); SGO (*n* = 272). Outliers and extreme values are included in the analyses but not shown in the figure. Statistically significant differences are indicated by asterisks: ^∗∗^*P* < 0.01 and ^∗∗∗^*P* < 0.001.

**Figure 3:**
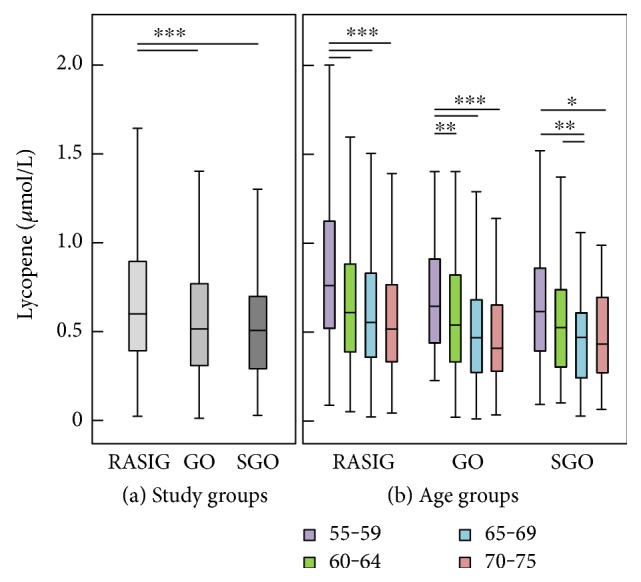
Lycopene concentration by study groups (a) and age groups (b). RASIG (*n* = 794); GO (*n* = 493); SGO (*n* = 272). Outliers and extreme values are included in the analyses but not shown in the figure. Statistically significant differences are indicated by asterisks: ^∗^*P* < 0.05, ^∗∗^*P* < 0.01, and ^∗∗∗^*P* < 0.001.

Biomarker concentrations differed significantly between study groups (Table 2 and Figures 1, 2, and 3). It is noteworthy that GO and SGO differed only in uric acid with significantly higher concentrations in the SGO group. Cysteine was highest in GO, glutathione in SGO, *α*-tocopherol was highest in both GO and SGO while lycopene was lower in GO and SGO than in the RASIG group. Interestingly, GO differed in all biomarkers from the RASIG group except in uric acid and total glutathione (Table 2). In detail, GO had significantly higher concentrations of ascorbic acid, total free cysteine, *α*-tocopherol, and 3-nitrotyrosine and lower concentrations of lycopene, protein carbonyls, and malondialdehyde. Furthermore, SGO were different from RASIG in all biomarkers except for uric acid, 3-nitrotyrosine, and malondialdehyde with higher concentrations of ascorbic acid, total free cysteine, total glutathione, and *α*-tocopherol and lower concentrations of protein carbonyls and lycopene.

We performed a univariate general linear model adjusted for age, BMI, gender, smoking status, and country to assess whether these differences were still present after adjustment. This was true for glutathione and *α*-tocopherol, as well as for all three oxidative stress biomarkers (protein carbonyls, 3-nitrotyrosine, and malondialdehyde), although lycopene (*P* < 0.069) and cysteine (*P* < 0.072) also reached the borderline of significance.

The comparison of biomarker concentrations of our study groups with a middle-aged control group is shown in Table 3. In general, the older groups revealed significantly different antioxidant concentrations compared to the reference group. The RASIG, GO, and SGO groups had significantly higher mean uric acid, cysteine, and *α*-tocopherol and lower lycopene compared to the reference group. There were no differences in terms of oxidative stress biomarkers between the reference group and RASIG aged 55–75 years. Interestingly, while the GO and SGO groups had significantly lower protein carbonyl concentrations compared to the reference group, only the GO group had higher 3-nitrotyrosine compared to the reference group.

Table 4 shows the correlation coefficients among the assessed biomarkers in all participants aged ≥55 years. The highest correlation coefficients were found between malondialdehyde and protein carbonyls (*r* = 0.322), malondialdehyde and ascorbic acid (*r* = −0.240), followed by protein carbonyls and *α*-tocopherol (*r* = −0.193). A positive correlation was observed between the antioxidants ascorbic acid and *α*-tocopherol (*r* = 0.164). When these correlations were performed only in the RASIG group, the direction and strength of correlations were similar (results not shown).

The correlations of the individual biomarkers with age are shown in Table 5. Significant positive correlation coefficients were observed for uric acid, cysteine, and 3-nitrotyrosine while a significant inverse association was only seen for lycopene. A weak significant positive correlation between protein carbonyls and age was only observed in the RASIG group (*r* = 0.098; *P* < 0.01, data not shown).

In a final multiple regression model with a forward approach, we aimed to identify independent biomarkers with the highest correlation with age (Table 6). Therefore, all biomarkers were assessed as covariates. Confirming the correlations from Table 5, uric acid, cysteine, 3-nitrotyrosine, and lycopene were significantly associated with age, and also, protein carbonyls remained in the model with a positive association.

## 4. Discussion

In general, the older groups revealed higher antioxidant (uric acid, cysteine, and *α*-tocopherol) and lower lycopene concentrations compared to the reference group. Lycopene, total cysteine, uric acid, protein carbonyls, and 3-nitrotyrosine were significantly and independently associated with age in the multiple linear regression model among participants aged 55–75 years. Higher cysteine and *α*-tocopherol, but lower lycopene in both GO and SGO compared to RASIG seem to be associated with a “beneficial” lifestyle, while the significantly lower malondialdehyde and higher 3-nitrotyrosine only in the GO compared to the RASIG group may indicate that families with long-living members are genetically better equipped to handle oxidative stress, yet the cause and impact of the higher 3-nitrotyrosine levels remain unclear.

We assume that (1) if there are differences between RASIG and the other two groups but no differences between GO and SGO, the reason may be lifestyle-related and (2) differences between GO and SGO (irrespective of the RASIG results) indicate genetic reasons.

Applying these criteria to the biomarkers assessed in the present study leads to the conclusion that after adjustment for age, BMI, smoking status, gender, and country, the differences in protein carbonyls, malondialdehyde, 3-nitrotyrosine, *α*-tocopherol, cysteine, and glutathione between study groups seem to be lifestyle-related whereas genetics seem to play a minor role. For the case where both GO and SGO differ to the control group (protein carbonyls, lycopene, and *α*-tocopherol), this might indicate the influence of lifestyle. Thus, our results show that lifestyle is an important contributor to redox biomarkers.

One might hypothesize that when GO differ to the other two groups and the reference group, this difference may be attributed to genetics. This was the case for 3-nitrotyrosine, cysteine (both higher than the other groups), and malondialdehyde (lower than the other groups). In our study, there was no significant difference between GO and SGO groups for these three biomarkers, yet a clear tendency exists according to the data in Tables 2 and 3. Concentrations of malondialdehyde, 3-nitrotyrosine, and total cysteine were still different between the GO and the RASIG groups after the GLM adjustment, which may be an indication of a genetic contribution in age-associated handling of oxidative stress.

Direct comparison of the study groups revealed that the GO group had significantly lower concentrations of protein carbonyls and malondialdehyde accompanied by higher concentrations of cysteine, ascorbic acid, and *α*-tocopherol compared to RASIG. Contrarily, GO had lower lycopene and higher 3-nitrotyrosine than the RASIG group. These differences may be due to better metabolic profiles or due to a generally healthier nutrient intake, despite less processed tomato products which are especially rich in lycopene.

It is widely accepted that there is a relationship between the aging process and oxidative stress; however, most studies leading to this theory have been carried out in model systems and only few studies have analyzed different biomarkers of oxidative stress in healthy humans of various age groups [3, 6].

Protein carbonyls are considered to be relatively stable [23] and early markers [24] of oxidative stress. Measuring carbonylation of plasma proteins enables evaluating the global oxidation status in plasma. Protein carbonyls have been analyzed in a multitude of studies ranging from cell culture, animal, to human studies and are related to the aging process. They have been shown to be a predictor of mortality in moderately to severely disabled older women [10]. Komosinska-Vassev et al. showed a positive correlation of protein carbonyls with age in a study of 56 men and women aged ≥55 years (*r* = 0.52) [25]. Other authors also found a strong positive correlation of protein carbonyls with age (*r* = 0.786) in 80 participants between the age of 18 and 85 [6], whereas in the present study, the correlation for the age range of 55–75 was very weak (*r* = 0.098) but still statistically significant. Cakatay et al. demonstrated that plasma protein carbonyl levels of elderly participants were significantly higher in comparison to those of middle-aged and young participants [26]. In the present study, protein carbonyls only correlated with age in the RASIG group (*r* = 0.098, *P* < 0.01; data not shown) but not when all three study groups were combined. Most interestingly, the RASIG group had statistically significantly higher protein carbonyl concentrations than the GO as well as the SGO while there was no difference between the GO and SGO. After adjusting for covariates (including smoking status), this difference remained.

For 3-nitrotyrosine, we observed a weak, positive association with age. In contrast to protein carbonyls, 3-nitrotyrosine was significantly higher in GO compared to RASIG. 3-Nitrotyrosine has been described to be a stable marker of oxidative/nitrative stress in some inflammatory diseases [27]. It arises from nitration involving reactive nitrogen species (RNS) and peroxidase-mediated nitrite oxidation. Today, myeloperoxidase is considered to be involved in this pathway. Thus, 3-nitrotyrosine may play a role in inflammation rather than the aging process itself [28]. Frijhoff et al. question whether 3-nitrotyrosine is clinically useful, in comparison to the already established markers of inflammatory processes such as C-reactive protein [29]. Plasma 3-nitrotyrosine levels in patients treated with anti-inflammatory drugs have been shown to decrease [30, 31]. This is one more hint why 3-nitrotyrosine may be a better marker for inflammation than for the aging process in general. Although the nitration of tyrosine residues on proteins can result in a loss of function [32], some authors have reported a gain of function [33]. Perhaps site-specific nitration may have a protective function or a role in longevity. In our study population, there might have been no dramatic difference of 3-nitrotyrosine in the different age groups because the participants were generally healthy. Only 4.0% of our participants had CRP concentrations ≥ 10 mg/L. Nevertheless, we checked the correlation between CRP and 3-nitrotyrosine but there was no correlation, neither in all participants nor in those with a high CRP level. Thus, the usefulness of 3-nitrotyrosine as a biomarker to evaluate oxidative stress remains to be elucidated.

Few large epidemiological studies have analyzed malondialdehyde. Block et al. suggest plasma malondialdehyde should be considered for most epidemiologic research on redox biomarkers [34] since they observed in a validation study that this biomarker had a good day-to-day stability. They propose malondialdehyde to be an effective marker of oxidative stress and state that the use of a single measure of malondialdehyde resulted in little attenuation [34]. In another study, the same authors found that malondialdehyde was not associated with age in 298 participants aged 18–78 years [35]. These results are supported by our finding that malondialdehyde was not associated with age in any of the three study groups. However, the GO group had significantly lower plasma malondialdehyde concentrations than RASIG. This difference remained even after adjusting for age, BMI, smoking status, gender, and country.

To counteract oxidative stress, there exist a network of antioxidant defense mechanisms. One of these antioxidants is the tripeptide glutathione (γ-glutamylcysteinylglycine). A decline of glutathione with age has previously been suggested in humans [36], and it has been demonstrated that the correlation of thiol groups in plasma with age was inverse (*r* = −0.718) [6].

Concentrations of glutathione and cysteine were lower in healthy old (mean age 70.3 years) than in middle-aged (mean age 39.8 years) participants (*n* = 8 each) [37].

We did not observe any correlation between glutathione and age. One explanation may be that we measured total glutathione instead of GSH/GSSH which is considered a better marker of the redox state. Giustarini et al. found an inverse correlation of glutathione with age but no correlation of cysteine with age in 41 participants [38]. Similarly, Jones et al. used plasma of 122 healthy individuals aged 19–85 years to analyze thiol-based redox changes [39]. They suggest that the capacity of the glutathione antioxidant system is maintained until 45 years and then declines rapidly. For the present analyses, participants were selected which were ≥55 years, yet we were unable to show a correlation between glutathione and age. In contrast to previous findings, cysteine is higher in the higher age groups in the RASIG and GO study groups and correlates positively with age among all participants (*r* = 0.152, *P* < 0.001).

It is assumed that lower glutathione concentrations occurring during aging and in different diseases may more likely result from low cysteine concentrations rather than due to oxidation since cysteine is the rate-limiting precursor of glutathione. Free cysteine is one of the main nonprotein thiols in plasma [40, 41] and considered a semiessential amino acid, since it must be taken up or synthesized from the essential amino acid methionine [42]. It is able to regulate nutrient metabolism, oxidative stress, physiologic signaling pathways, and associated diseases through the production of glutathione, hydrogen sulfide, and taurine [43]. An oral intervention with cysteine (as N-acetylcysteine) and glycine for 14 days resulted in significantly increased glutathione concentrations [37]. This was also true when only cysteine was supplemented, resulting in an increase in hepatic glutathione synthesis [43]. It is likely that the glutathione concentrations in our cross-sectional study were similar in the different age groups because cysteine may not have been limited. Previous results also suggest that the participants in our study had sufficient cysteine and thus glutathione levels [38]. Nevertheless, the requirement of cysteine may be elevated after oxidative events due to the consumption of glutathione. Some future research should clarify the role of cysteine in aging and as a dietary precursor of glutathione. Furthermore, it should be noted that some authors measured glutathione/thiol groups in plasma [6]. Glutathione is known as the most powerful cellular antioxidant, that is, present in red blood cells, and consequently, we analyzed glutathione in whole blood.

Concerning uric acid, our results show that there was no difference between RASIG and GO but higher concentrations in the SGO group than in the GO. However, adjusting for age, BMI, gender, smoking status, and country, these differences did not remain. Uric acid is derived from the degradation of purine nucleotides which can be of dietary or endogenous origin. Therefore, an increased intake of animal products or legumes/pulses in the SGO group is possible. There is a controversial discussion whether the positive effects of uric acid as an antioxidant are outweighed by its adverse effects concerning gout, coronary artery disease, hypertension, and stroke (for review, see [44]).

Besides endogenous antioxidants such as glutathione, cysteine, and uric acid, exogenous antioxidants are also required to counteract oxidative stress. Some of the most powerful antioxidants are of dietary origin such as ascorbic acid, *α*-tocopherol, lycopene, and other carotenoids. It is known that a high intake of fruits and vegetables is associated with a high plasma concentration of ascorbic acid [45]. Simultaneously, a diet rich in fruits and vegetables is associated with a reduced risk of some diseases such as CVD and cancers [46]. *α*-Tocopherol and ascorbic acid act synergistically in counteracting free radicals. *α*-Tocopherol is able to quench free radicals in a hydrophobic environment, for example, to terminate lipid peroxidation and the resulting tocopherol radical is then recycled by ascorbic acid [47]. We found an inverse correlation between *α*-tocopherol and malondialdehyde, an inverse association between ascorbic acid and malondialdehyde (all *P* < 0.001), and a positive correlation between *α*-tocopherol and ascorbic acid. These results are in accordance with the assumption that these antioxidants act synergistically.

Since these are diet-derived antioxidants and we did not adjust our models for dietary intake of fruit and vegetables, we cannot exclude that the diet or the season had an effect on the plasma concentration of these micronutrients. In a recent study, we have shown over the whole age-range of the MARK-AGE project from 35 to 75 (RASIG group) that lycopene was lower in higher age groups and this effect was independent of season [22]. It remains unclear if these differences observed in the older age groups result from a reduced intake, reduced absorption, increased storage in adipose tissue, or elevated degradation of lycopene.

The strengths of the present study include the large sample size and that all biomarkers described here were measured in one single laboratory in blinded form (samples from study groups and countries were in mixed random order by the biobank). For lipid-soluble micronutrient analysis, we have recently published the interbatch coefficients of variation which were 7.6% for lycopene and 6.3% for *α*-tocopherol in the MARK-AGE cohort [22]. Concerning the validity of the methods used for 3-nitrotyrosine analysis, we have previously reported good specificity, reproducibility, and accuracy for this in-house ELISA [48]. The ELISA method used here to analyze protein carbonyls has been validated in a multicenter ring study [49]. It was shown that carbonyl concentrations from three out of four laboratories participating in the ring study fell within 95% confidence intervals. Additionally, protein concentration was measured before both ELISAs and plasma samples were diluted to the same protein concentration. Standards for protein carbonyl ELISA were prepared according to Buss et al. [21] and run on every 96-well plate.

In terms of comparability to other studies, there is an excellent review from Giustarini et al. [50]. As the authors demonstrate, there exist numerous analytical methods to analyze protein carbonyls, malondialdehyde, GSH, tocopherols, and lycopene, among others. An overview is given for different derivatization and detection methods, health conditions, and units, demonstrating huge variety. For instance for malondialdehyde, Giustarini et al. showed that authors using methods comparable to ours (TBA derivatization and HPLC separation) published concentrations between 0.44 and 6.8 *μ*mol/L for plasma samples [50]. One reason for this variation may be that heparin plasma was used in some cases. Especially for malondialdehyde, it is important to measure this marker in EDTA plasma since lipid peroxidation can continue in serum and heparin plasma, thus artificially contributing to elevated malondialdehyde concentrations. EDTA and citrate can complex iron thus preventing Fenton reaction leading to lipid peroxidation.

For GSH and cysteine, the authors also report different methods (e.g., enzymatic, HPLC coupled with UV or fluorescence detection) and mean concentrations in whole blood, plasma, and erythrocytes for healthy and diseased individuals, which span over one to two orders of magnitude within the kind of used specimen; however, total GSH levels in the present study are in the upper range of previously reported mean whole blood values for healthy individuals measured by HPLC or recycling spectrophotometry (using Ellman's reagent).

This study has some limitations which must be mentioned. Since this study was observational, we cannot make statements on the changes of these biomarkers with age; therefore studies with repeated measurements and/or follow-up are needed. Furthermore our results may be specific for European/Caucasian subjects and thus not transferrable to other countries.

The inclusion of three different study groups with a large sample size (*n* = 1559) is one feature that distinguishes this study from others. Samples were collected and distributed in a blinded form to guarantee unbiased measurement and interpretation, and all analyses of biomarkers described here were carried out in one single laboratory, which significantly reduces interlaboratory variations. The assessment of different specific cellular and plasma biomarkers, that is, markers for oxidative damage together with antioxidants that are not analyzed by commercial kits but by in-house methods is certainly an important strength.

## 5. Conclusion

Here, we have provided an overview of the levels of the different redox biomarkers in human plasma and whole blood in three different study groups of the MARK-AGE project and their correlation with age. Interestingly, from all antioxidants measured, only lycopene was lower in the three aged groups. In addition, from the oxidative stress biomarkers, only 3-nitrotyrosine was increased in the descendants from long-living families compared to the control group, while the aged general population did not exhibit any difference compared to the middle-aged controls. Higher cysteine and *α*-tocopherol, but lower lycopene in both GO and SGO, compared to the RASIG seem to be associated with a “beneficial” lifestyle, while the significantly lower malondialdehyde and higher 3-nitrotyrosine which were observed only in the GO compared to the RASIG group may indicate that families with long-living members are genetically better equipped to handle oxidative stress. Thus, our present study suggests that age, lifestyle, and genetics could contribute to an individual's oxidative stress status.

## Figures and Tables

**Table 1:** Characteristics of the three study groups.

	All (*n* = 1559)	RASIG (*n* = 794)	GO (*n* = 493)	SGO (*n* = 272)	*P*
*Age (years)*	64.3 ± 5.4	64.5 ± 5.8	64.3 ± 4.9	63.9 ± 4.7	0.230
55–59 years (*n*, (%))	345 (22.1)	193 (24.3)	94 (19.1)	58 (21.3)	**<0.001**
60–64 years (*n*, (%))	448 (28.7)	210 (26.4)	149 (30.2)	89 (32.7)	
65–69 years (*n*, (%))	465 (29.8)	204 (25.7)	166 (33.7)	95 (34.9)	
70–75 years (*n*, (%))	301 (19.3)	187 (23.6)	84 (17.0)	30 (11.0)	
Gender, male (*n*, (%))	743 (47.7)	386 (48.6)	213 (43.2)	144 (52.9)	**0.027**
Smoker, current (*n*, (%))	231 (14.8)	142 (17.9)	63 (12.6)	27 (9.9)	**0.001**
*BMI (kg/m^2^)*	27.3 ± 4.5	27.6 ± 4.7	26.6 ± 4.4	27.3 ± 4.2	0.692
<25 (*n*, (%))	516 (33.1)	249 (31.4)	188 (38.1)	79 (29.0)	**0.036**
25 to <30 (*n*, (%))	673 (43.2)	343 (43.3)	199 (40.4)	131 (48.2)	
≥30 (*n*, (%))	369 (23.7)	201 (25.3)	106 (21.5)	62 (22.8)	
*Country*					
Belgium (*n*, (%))	472 (30.3)	155 (19.5)	190 (38.5)	127 (46.7)	**<0.001**
Finland (*n*, (%))	253 (16.2)	69 (8.7)	132 (26.8)	52 (19.1)	
Greece (*n*, (%))	209 (13.4)	187 (23.6)	18 (3.7)	4 (1.5)	
Italy (*n*, (%))	323 (20.7)	187 (23.6)	87 (17.6)	49 (18.0)	
Poland (*n*, (%))	302 (19.4)	196 (24.7)	66 (13.4)	40 (14.7)	

Values are means ± SD; *P* value: one-way ANOVA (continuous variables) and Pearson's chi-squared test (prevalence).

**Table 2:** Biomarker concentrations in the three study populations^1^.

	All (*n* = 1559)	RASIG (*n* = 794)	GO (*n* = 493)	SGO (*n* = 272)	*P*
*Antioxidants*					
Ascorbic acid (mg/L)	4.41 (4.26; 4.58)	4.13 (3.91; 4.36)	4.73 (4.44; 5.02)^‡^	4.70 (4.33; 5.08)^‡^	**0.002**
*Adjusted GLM^2^*		4.32 (4.13; 4.52)	4.17 (3.87; 4.49)	3.85 (3.32; 4.41)	0.258
Uric acid (mg/L)	45.9 (45.3; 46.5)	45.7 (44.9; 46.5)	45.3 (44.2; 46.3)	47.4 (46.0; 48.8)^◊^	**0.047**
*Adjusted GLM^2^*		45.4 (44.6; 46.1)	46.0 (44.7; 47.2)	47.0 (44.7; 49.3)	0.341
Total cysteine (*μ*mol/L)	148.2 (146.6; 149.8)	144.8 (142.5; 147.1)	150.8 (147.9; 153.6)^‡^	153.3 (149.3; 157.3)^‡^	**<0.001**
*Adjusted GLM^2^*		144.2 (141.8; 146.6)	149.6 (145.7; 153.4)^‡^	145.5 (138.4; 152.6)	0.072
Total glutathione (*μ*mol/L)	1100 (1091; 1110)	1088 (1074; 1102)	1110 (1093; 1127)	1120 (1099; 1141)^‡^	**0.024**
*Adjusted GLM^2^*		1092 (1077; 1106)	1112 (1089; 1135)	1149 (1106; 1193)^‡^	**0.025**
Lycopene (*μ*mol/L)	0.574 (0.556; 0.592)	0.631 (0.604; 0.659)	0.525 (0.497; 0.554)^‡^	0.503 (0.464; 0.543)^‡^	**<0.001**
*Adjusted GLM^2^*		0.608 (0.583; 0.635)	0.566 (0.526; 0.606)	0.539 (0.470; 0.613)	0.069
*α*-Tocopherol (*μ*mol/L)	28.8 (28.4; 29.2)	27.8 (27.3; 28.3)	30.1 (29.4; 30.8)^‡^	29.6 (28.6; 30.5)^‡^	**<0.001**
*Adjusted GLM^2^*		27.9 (27.4; 28.5)	29.3 (28.4; 30.3)^‡^	29.9 (28.2; 31.7)^‡^	**0.005**
*Oxidative stress biomarkers*					
Protein Carbonyls (nmol/mg)	0.577 (0.573; 0.582)	0.595 (0.590; 0.601)	0.558 (0.551; 0.566)^‡^	0.561 (0.552; 0.571)^‡^	**<0.001**
*Adjusted GLM^2^*		0.591 (0.585; 0.597)	0.566 (0.557; 0.575)^‡^	0.564 (0.547; 0.581)^‡^	**<0.001**
3-Nitrotyrosine (pmol/mg)	4.11 (3.97; 4.25)	3.94 (3.75; 4.13)	4.40 (4.14; 4.67)^‡^	4.10 (3.77; 4.44)	**0.018**
*Adjusted GLM^2^*		3.90 (3.70; 4.11)	4.38 (4.03; 4.74)^‡^	3.76 (3.18; 4.38)	**0.048**
Malondialdehyde (*μ*mol/L)	0.316 (0.306; 0.326)	0.336 (0.322; 0.350)	0.284 (0.266; 0.302)^‡^	0.317 (0.290; 0.346)	**<0.001**
*Adjusted GLM^2^*		0.334 (0.319; 0.348)	0.288 (0.267; 0.311)^‡^	0.314 (0.274; 0.357)	**0.004**

^1^Geometric mean (95% CI). ^2^Adjusted general linear model (GLM): univariate general linear model adjusted for age, BMI, smoking status, gender, and country (center). ^‡^Statistically significant difference to RASIG. ^◊^Statistically significant difference to GO. *P* values: statistically significant differences were determined by one-way ANOVA with Tukey's post hoc test and by Fisher's least significant difference post hoc test in the GLM.

**Table 3:** Comparison of study groups with a reference group.

	Reference Group (RASIG; 35–54 years) (*n* = 683)	RASIG (55–75 years) (*n* = 794)	GO (55–75 years) (*n* = 493)	SGO (55–75 years) (*n* = 272)
*Antioxidants*				
Ascorbic acid (mg/L)	4.37 (4.12; 4.63)	4.13 (3.91; 4.36)	4.73 (4.44; 5.02)	4.70 (4.33; 5.08)
Uric acid (mg/L)	42.4 (41.6; 43.3)	**45.7 (44.9; 46.5)** ^∗∗∗^	**45.3 (44.2; 46.3)** ^∗∗∗^	**47.4 (46.0; 48.8)** ^∗∗∗^
Total cysteine (*μ*mol/L)	130.3 (128.1; 132.5)	**143.0 (140.7; 145.3)** ^∗∗∗^	**149.0 (146.1; 151.9)** ^∗∗∗^	**151.4 (147.4; 155.5)** ^∗∗∗^
Total glutathione (*μ*mol/L)	1107 (1092; 1121)	1088 (1074; 1101)	1110 (1093; 1127)	1120 (1099; 1142)
Lycopene (*μ*mol/L)	0.83 (0.80; 0.86)	**0.63 (0.60; 0.66)** ^∗∗∗^	**0.53 (0.50; 0.55)** ^∗∗∗^	**0.50 (0.46; 0.54)** ^∗∗∗^
*α*-Tocopherol (*μ*mol/L)	25.7 (10.4; 10.6)	**27.8 (10.9; 11.2)** ^∗∗∗^	**30.1 (11.4; 11.7)** ^∗∗∗^	**29.6 (11.2; 11.7)** ^∗∗∗^
*Oxidative stress biomarkers*				
Protein carbonyls (nmol/mg)	0.603 (0.598; 0.609)	0.595 (0.590; 0.601)	**0.558 (0.551; 0.566)** ^∗∗∗^	**0.561 (0.552; 0.571)** ^∗∗∗^
3-Nitrotyrosine (pmol/mg)	3.8 (3.6; 4.0)	3.9 (3.8; 4.1)	**4.4 (4.1; 4.7)** ^∗∗^	4.1 (3.8; 4.4)
Malondialdehyde (*μ*mol/L)	0.31 (0.30; 0.32)	0.34 (0.32; 0.35)	0.28 (0.27; 0.30)	0.32 (0.29; 0.35)

^∗∗∗^ *P* < 0.001 and ^∗∗^*P* < 0.01 by one-way ANOVA with Tukey's post hoc test.

**Table 4:** Correlations between biomarkers in all three study groups in participants aged ≥55 years^1^.

	Ascorbic acid	Uric acid	Total cysteine	Total glutathione	Lycopene	*α*-Tocopherol	Protein carbonyls	3-Nitro-tyrosine	Malondialdehyde
Ascorbic acid		**−0.083** ^∗∗∗^	**0.140** ^∗∗∗^	0.030	**−0.103** ^∗∗∗^	**0.164** ^∗∗∗^	**−0.157** ^∗∗∗^	−0.027	**−0.240** ^∗∗∗^
Uric acid			**0.067** ^∗∗^	−0.021	**−0.099** ^∗∗∗^	**0.059** ^∗^	−0.020	**−0.054** ^∗^	0.019
Total cysteine				0.047	**−0.053** ^∗^	**0.103** ^∗∗∗^	**0.062** ^∗^	0.043	−0.034
Total glutathione					0.032	−0.033	−0.007	−0.015	0.030
Lycopene						**0.073** ^∗∗^	**0.088** ^∗∗∗^	**−0.092** ^∗∗∗^	**0.159** ^∗∗∗^
*α*-Tocopherol							**−0.193** ^∗∗∗^	**0.062** ^∗^	**−0.132** ^∗∗∗^
Protein carbonyls								0.001	**0.322** ^∗∗∗^
3-Nitrotyrosine									0.019
Malondialdehyde									

^1^Pearson correlation coefficient *r*. Statistically significant correlations are marked by ^∗^*P* < 0.05, ^∗∗^*P* < 0.01, and ^∗∗∗^*P* < 0.001. (*n* = 1559).

**Table 5:** Correlations between biomarkers and age among all participants (aged ≥55 years)^1^.

	*r*	*P*
Ascorbic acid	0.026	0.297
Uric acid	**0.092**	**<0.001**
Total cysteine	**0.152**	**<0.001**
Total glutathione	−0.035	0.167
Lycopene	**−0.224**	**<0.001**
*α*-Tocopherol	−0.005	0.847
Protein carbonyls	0.036	0.158
3-Nitrotyrosine	**0.066**	**0.009**
Malondialdehyde	−0.037	0.146

^1^Pearson correlation coefficient *r* (*n* = 1559).

**Table 6:** Associations of oxidative stress markers and antioxidants with age^1^.

Compound	*B*	95% CI	*r*	*r^2^*	*P*
(Constant)	59.13	56.79, 61.48			**<0.001**
Lycopene (*μ*mol/L)	−2.783	−3.440, −2.126	−0.207	0.047	**<0.001**
Total cysteine (mol/L)	0.021	0.013, 0.029	0.133	0.020	**<0.001**
Uric acid (mg/L)	0.031	0.009, 0.054	0.071	0.004	**0.005**
Protein carbonyls (nmol/mg)	3.274	0.252, 6.296	0.054	0.003	**0.034**
3-Nitrotyrosine (pmol/mg)	0.094	0.004, 0.183	0.052	0.003	**0.040**

^1^Multiple linear regression analysis with a forward approach to identify independent blood biomarkers with highest correlation to age; all biomarkers including ascorbic acid, glutathione, malondialdehyde, and *α*-tocopherol were assessed as covariates in the initial model; partial *r* and *r^2^*; *R* = 0.277, *R*^2^ = 0.077 (*n* = 1545).

## Abbreviations

ELISA: Enzyme-linked immunosorbent assay
GEHA: Genetics of healthy aging
GLM: General linear model
GSH: Glutathione
GO: GEHA offspring
HPLC: High-performance liquid chromatography
RASIG: Recruited from the age-stratified general population
SGO: Spouses of GO.
